# Soft Tissue Conduction Activates the Auditory Pathway in the Brain

**DOI:** 10.3390/audiolres14010018

**Published:** 2024-02-16

**Authors:** Miriam Geal-Dor, Haim Sohmer

**Affiliations:** 1 Speech & Hearing Center, Hadassah Hebrew University Medical Center, Jerusalem 91200, Israel; 2 Department of Communication Disorders, Hadassah Academic College, Jerusalem 91200, Israel; 3 Department of Medical Neurobiology (Physiology), Hebrew University-Hadassah Medical School, P.O. Box 12272, Jerusalem 91120, Israel; haims@ekmd.huji.ac.il

**Keywords:** soft tissue conduction, speech recognition, auditory pathway, cutaneous mechanoreceptors, somatosensory pathway

## Abstract

Soft tissue conduction is a mode of hearing which differs from air and bone conduction since the soft tissues of the body convey the audio-frequency vibrations to the ear. It is elicited by inducing soft tissue vibrations with an external vibrator applied to sites on the body or by intrinsic vibrations resulting from vocalization or the heartbeat. However, the same external vibrator applied to the skin sites also excites cutaneous mechanoreceptors, and attempts have been made to assist patients with hearing loss by audio–tactile substitution. The present study was conducted to assess the contribution of the auditory nerve and brainstem pathways to soft tissue conduction hearing. The study involved 20 normal hearing students, equipped with ear plugs to reduce the possibility of their response to air-conducted sounds produced by the external vibrator. Pure tone audiograms and speech reception (recognition) thresholds were determined in response to the delivery of the stimuli by a clinical bone vibrator applied to the cheek, neck and shoulder. Pure tone and speech recognition thresholds were obtained; the participants were able to repeat the words they heard by soft tissue conduction, confirming that the auditory pathways in the brain had been stimulated, with minimal involvement of the somatosensory pathways.

## 1. Introduction

Soft tissue conduction (STC) is a mode of hearing which is distinct from air conduction (AC), in which vibrations of the tympanic membrane, the middle ear ossicles and the windows into the inner ear are induced. It also differs from bone conduction (BC) hearing, which is based on the induction of actual vibrations of skull bone, and is always elicited by an external vibrator, such as a clinical vibrator or bone-anchored hearing aid (BAHA). The skull vibrations involve the bony parts of the outer, middle and inner ears. In STC hearing, the physical medium through which the vibrations are conducted to the ear is by the soft tissues of the body. STC is a more natural mode of hearing that we meet in everyday life and not only in the clinic. STC can be elicited by inducing vibrations of the soft tissues of the body, such as by applying an external vibrator (e.g., a clinical bone vibrator) to the surface of the body (the skin) [1,2,3]. “Distantly-presented bone conduction perception” [3] delivered to body sites on the neck, the clavicle and the upper limbs can also be considered a form of STC stimulation. STC hearing can even be elicited by intrinsic, natural body vibrations, such as those accompanying vocalizations [4,5], the heartbeat [6] or the hearing of maternal sounds by the fetus in utero. Further expressions of STC hearing include body conduction [7] (which is defined as the acoustic noise produced by functional magnetic resonance imaging (_f_MRI) equipment, remaining in the presence of a head helmet, with external ear protectors or earmuffs and also earplugs, or all of them together) and ankle audiometry [8]. Ankle audiometry [8] represents an extension of an earlier study [9] in which hearing was assessed in response to the delivery of vibratory stimuli by a mini shaker to several sites over the body, including the leg, in superior semi-circular canal dehiscence patients. This is a rare condition in patients who are highly sensitive to sound, suffer from sound-induced vertigo and hear their own voice, heartbeat and chewing. It usually results from an opening (a dehiscence) in the superior semi-circular canal of the inner ear, providing communication between the cerebrospinal fluid in the cranial cavity and inner ear fluid. Normal hearing control subjects served as control subjects. The patients and the control subjects were equipped with earplugs in order to block their hearing of the stimuli by AC hearing. In the present report, the general term “soft tissue conduction” will be used collectively to describe these forms of hearing. In each of these examples, it is thought that the vibrations induced by the external vibrator or by the intrinsic vibrations (e.g., heartbeat, vocal cords) are conducted by the soft tissues of the body to the ear, where they excite the inner ear hair cells and auditory nerve fibers, eliciting hearing. The fact that the soft tissues of the body are able to conduct vibrations is demonstrated by the use of a stethoscope by the clinician. The vibrations induced in the body by the heartbeat, blood flow in the major vessels, pulmonary air flow and even intestinal movements are conveyed by the soft tissues to the surface of the body (skin). By applying his stethoscope to the skin, the clinician can assess the function of the relevant organs. When STC is elicited by applying an external vibrator (a clinical bone vibrator or a mini shaker) to sites on the body, it is mandatory to provide adequate controls to ensure that the subjects are not responding to the AC sounds produced inadvertently by the external stimulator in the course of its delivering vibrations to the skin. This is particularly relevant to those studies in which the vibratory stimuli are delivered to sites on the body more distant from the ear. In such applications, higher-intensity stimuli are then required in order to reach the behavioral threshold of the participants [2,3,8,9]. In other words, the vibrations induced at a site on the body are progressively reduced in magnitude as they are conducted along the soft tissues toward the ear; this is probably a reflection of the inverse square law: the magnitude of a physical phenomenon is inversely proportional to the distance. In such cases, there is thus a “trade off” between distance and intensity, so that the greater the distance of the stimulation site from the ear, the greater the stimulus intensity required to reach the behavioral threshold. There is then the possibility that the participants may respond to the AC sound component generated by the external stimulator, and not to the vibratory component of the stimulus. As an example of this possibility, it was reported that the normal hearing control subjects in the study of superior semi-circular canal dehiscence patients [8] also responded to the mini-shaker stimulation, though at 4 to 23 dB higher intensity stimulation than the superior semi-circular canal dehiscence patients. Therefore, it is possible that part of the responses in the control subjects may have been to the AC sound component.

In addition, when STC is induced by a vibrator applied to the skin of the body, it is obvious that the skin vibrations induced at the same site and time can excite cutaneous mechanoreceptors, especially the Meissner and Pacinian corpuscles, which are activated by the low-frequency mechanical vibrations (the maximal sensitivity of the Meissner corpuscles is 30–50 Hz; that of the Pacinian corpuscles is 200–350 Hz) [10]. Therefore, in studies involving STC stimulation in response to extrinsically induced body vibrations which are to be detected behaviorally by the subjects, there is the possibility that the subjects may “detect” the presence of the vibrations by means of their cutaneous mechanoreceptors and somatosensory pathway in the spinal cord, brainstem and cortex. This is especially relevant in light of the attempts to assist patients with hearing loss by the use of audio–tactile integration or substitution [11,12,13], that is, by delivering auditory stimuli coupled with tactile stimuli, making use of the communications between the somatosensory and auditory neural pathways.

The purposes of the present report are as follows: a—attempt to provide confirmation that STC hearing is not the result of the subjects responding behaviorally to the AC sounds produced by the stimulator; b—to assess the contribution of the auditory nerves and brain auditory pathways to STC hearing; c—the present report does not involve activation of the cutaneous mechanoreceptors of the somatosensory system when vibratory stimuli are delivered to the skin, together with the speech stimuli that require comprehension specific to the auditory pathway; and d—to contribute to a characterization of the nature of STC.

## 2. Methods

In a preliminary stage of this study, conducted on a small group of the participants, the stimulation sites on the skin and the types of auditory stimulation to be used were explored, together with attempts to reduce the possibility that they would respond to the AC components of the sound stimuli. In the final experiment, twenty female subjects recruited from a local college (mean ± standard deviation: age 25.8 ± 1.2 years; range 24 to 28 years) served as participants in the study. All participants were healthy, with no history of ear infections or ear operations, with a Body Mass Index (BMI) within normal limits. The female subjects were chosen after it was found that there is no effect of BMI in females on the correlation between the threshold and stimulation at more distant sites [2]. All of the participants had normal hearing, defined as AC thresholds at or better than 15 dB HL at the frequencies 0.5, 1.0, 2.0 and 4.0k Hz. All auditory testing was conducted using the same clinical audiometer (AudioStar Pro™, Grason Stadler Inc., Eden Prairie, MN, USA) in the same audiometric booth by the same two testers. The study was reviewed and approved (427-23) by the Hadassah Academic College Ethics Committee and written, informed consent was given by all participants.

The participants initially underwent a conventional audiometric AC threshold evaluation conducted with warble tones (as is standard at our facility) by the same two testers in the conventional manner (American National Standard Institute S3.31 1978, 1986), using the modified Hughson–Westlake technique.

The STC threshold was determined with 0.5k Hz, 1.0k Hz, 2.0k Hz and 4.0k Hz stimulation delivered by a clinical bone vibrator (Radioear B71, Radioear, New Eagle, PA, USA), applied using a bone vibrator metal head band (Radioear P3333, Radioear), to the skin at the three STC sites, the cheek (with an open mouth to ensure that there was no contact between the cheek and the teeth), neck (over the sternocleidomastoid muscle) and shoulder (over the lower trapezius muscle), with the standard application force of approximately 500 g (5 N). Following this, an attempt was also made to reach body sites further away from the ear, such as the arm, waist and thigh. However, the intensity required at these more distant sites to reach the threshold in response to STC stimulation was also heard via air conduction; therefore, these sites were not included in the final study. The head band was used with all participants, so that the application force would be similar in all. In addition, in three of the participants, the bone vibrator was also pressed to the same sites using a spring, calibrated to deliver a 5 N force to apply the stimuli, as in previous studies [2]. These three subjects responded to stimuli delivered by the spring and by the head band with the same threshold, and they reported that the application force with the spring and with the head band seemed to them to be identical.

The participants were equipped with earplugs (Classic SuperFit 30 AeroCo, E-A-R, Indianapolis, IN, USA) deeply inserted into each outer ear canal in order to reduce the possibility that the participants would respond to the air-conducted sound produced by the bone vibrator. Thresholds were also determined with the bone vibrator in the air, directly over, but not touching, the skin-cheek site, as an “in-air control”. The cheek was chosen as the “in-air control” site, since it was the site closest to the ear—the site at which the participants would be more likely to respond to any AC coming from the bone vibrator. If the threshold for stimulation at the “in-air control” site near the cheek was higher (worse) than the threshold at the different STC sites, then the “in-air control” would surely be worse than that at the other sites, even those more distant from the ear.

Using this experimental protocol, four threshold audiograms were conducted in each subject in response to stimulation delivered to the three STC sites (cheek, neck and shoulder: the lower trapezius muscle) and one with the bone vibrator in the air, about 1 cm above the cheek site, not in contact with it (“in-air control”). For each stimulation site, thresholds for warble tones at 0.5k Hz, 1.0k Hz, 2.0k Hz and 4.0k Hz, and also for speech reception (recognition) thresholds (SRT) to bisyllabic spondee words (the lowest intensity at which the subjects were able to accurately repeat over 50% of the words they heard) were determined as described [14]. The frequencies 0.5k Hz, 1.0k Hz, 2.0k Hz and 4k Hz were chosen because at these frequencies the audiometer and the bone vibrator stimulator were able to deliver these frequencies without output intensity limitations, and not at 0.125k Hz and 0.250k Hz as a result of loudness limitations at these frequencies. The stimulus intensities are given as dB audiometer instrument settings in the bone vibrator mode.

## 3. Results

**Figure 1:**
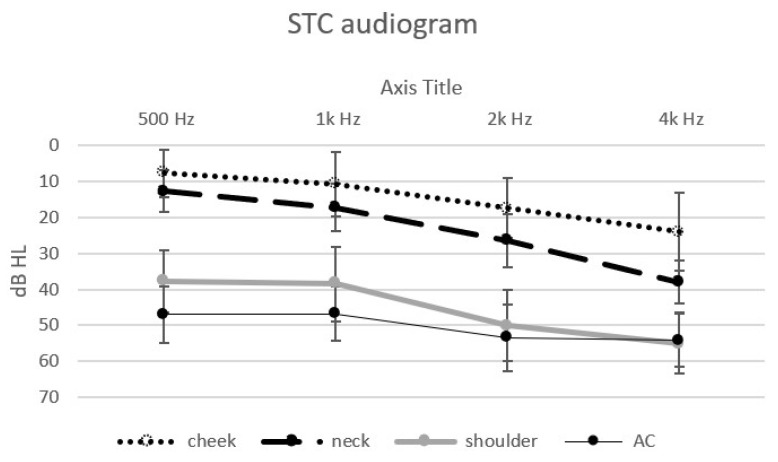
Mean threshold audiograms (±SD) in dB to the four tonal stimuli at the different sites: cheek—dotted line, neck—dashed line, shoulder—thick continuous line and “in-air control” (above the cheek)—thin continuous line (stimulus intensities in dB audiometer instrument settings in the bone vibrator mode).

**Figure 2:**
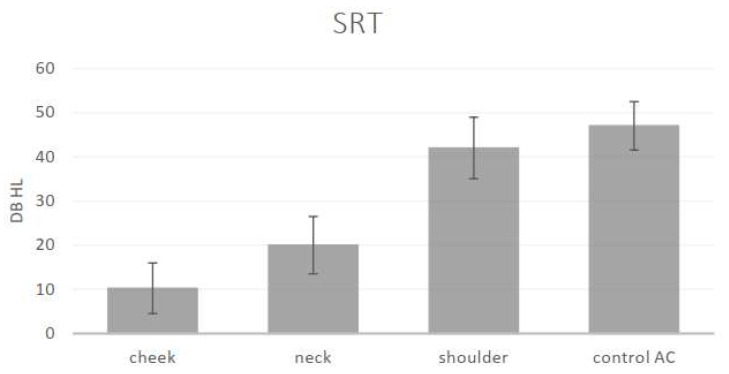
Means and standard deviations of the speech reception (recognition) thresholds (SRT) in dB for the different sites: cheek, neck, shoulder and “in-air control AC” (stimulus intensities in dB audiometer instrument settings in the bone vibrator mode).

Thresholds could be obtained from each of the 20 normal hearing participants, at each of the frequencies in response to the vibratory tonal stimuli delivered to the three STC sites (cheek, neck and shoulder), to the “in-air control” above the cheek and the speech reception thresholds. The mean threshold audiograms are displayed in Figure 1. The speech reception (recognition) thresholds and standard deviations are displayed in Figure 2.

One-way Analysis of Variance (ANOVA) revealed a significant effect of the stimulation site for each of the different stimuli: 500 Hz (F (3) = 131, *p* < 0.001), 1k Hz (F (3) = 77.65, *p* < 0.001), 2k Hz (F (3) = 87.66, *p* < 0.001), 4k Hz (F (3) = 57.54, *p* < 0.001) and SRT (F (3) = 160.48, *p* < 0.001). Post hoc analysis (Scheffe) revealed an effect of the distance of the stimulation site from the ear for the low frequencies: the thresholds for 500 Hz and 1k Hz at the cheek and neck sites were significantly lower (better) than those at the shoulder and were significantly lower (better) than the “in-air control” (above the cheek) threshold. The thresholds for the higher frequencies (2k Hz and 4k Hz) and the SRT were also significantly affected by distance. The threshold at the cheek site (closer to the ear) was significantly lower (better); on the neck, this threshold was slightly higher, and it was significantly the highest at the shoulder and for the “in-air (cheek) control”. The SRT at the cheek and neck were significantly lower (better) than that for the AC control. There was no significant difference between the thresholds for the higher frequencies (2k Hz, 4k Hz) at the shoulder and the “in-air control”. The differences between the sites show the effect of the distance of the STC stimulation site on the threshold: the greater the distance of the stimulation site from the ear, the higher the threshold.

Following this, the effect of the different stimulus frequencies was also assessed. A significant effect at the three stimulus sites was found: cheek (F (4) = 12.82, p < 0.001), neck (F (4) = 40.82, *p* < 0.001), shoulder (F (4) = 14.55, *p* < 0.001). There was no significant difference in the air control between the different stimuli thresholds. Post hoc analysis (Scheffe) revealed a difference between the lower frequencies, 500 Hz and 1k Hz (lower—better thresholds), and the thresholds for the higher frequencies, 2k Hz and 4k Hz, and the SRT.

The differences found with respect to the frequency of the stimuli show that STC stimulation is more effective (better at lower thresholds) at the lower frequencies than at the higher frequencies.

## 4. Discussion

The finding that the thresholds were higher at the stimulus sites more distant from the ear is similar to the results reported in many of the studies on STC [2,3,8,9,15]. These findings likely reflect “the inverse square law”: the magnitude of a physical phenomenon is inversely proportional to the distance, i.e., the magnitude of the vibrations induced at a site are progressively reduced as they are conducted toward the ear.

In addition, the result that the thresholds for the lower frequencies were lower (better) than those for the higher frequencies is similar to the results of other STC studies [16]. This result may lead to the suggestion that the STC pathway has its own specific frequency characteristics that are unique to the STC pathway and may contribute to the distinction between it from the other known hearing pathways of air conduction and bone conduction.

When STC is elicited by using a bone vibrator to deliver external vibratory stimulation to sites on the body, it is obvious that two sets of sensory receptors and neural-brain pathways were activated at the same time: the auditory and the somatosensory systems. With respect to the auditory system, confirmation is then required that the subjects, equipped with ear plugs, were not responding to their detection of the AC sound stimuli produced inadvertently at the same time by the bone vibrator. This was achieved in the present and other studies, not only with the use of ear plugs, but also by applying an “in-air control”, i.e., by holding the vibrator in the air directly over the cheek stimulation site, but not in contact with it, and not accepting responses reported by the subject in this situation. Pure tone threshold audiograms of the subjects were obtained, together with their ability to respond to (to repeat) the speech audiometry stimuli: speech reception (recognition) thresholds (SRT). These results, together with the difference limens (just noticeable differences) for the frequencies reported [3], provide conclusive evidence that their auditory nerves and brain pathways, including the auditory cortex, were stimulated. The result that the subjects comprehended the spoken words provides evidence that they heard and understood the words, and then their recitation (recognition and repetition of the words) provides convincing evidence that the auditory pathway and cortex are involved, and this is the major advantage and contribution of the present study. However, the somatosensory system was likely also activated at the same time by the low-frequency components of the speech stimuli delivered by the vibrator to sites on the body, though without activation of the auditory nerve and brain auditory pathway, as reported in [13]. A future study could also include the evaluation of responses to 125 and 250 Hz of vibratory stimulation (however, only after providing sufficient output intensity at these frequencies) in order to enable better comparison of the tactile and auditory systems. The audio–tactile integration or substitution studies which combine auditory with tactile stimulation [11,12,13] have led to the development of audio-to-tactile sensory substitution devices which transform the low-frequency components of speech signals into tactile vibrations delivered, for example, to the finger tips. The results of such studies showed immediate and robust improvement in speech recognition, and were able to enhance otherwise inaudible tones in the specific range of frequencies of the Pacinian corpuscles (200–350 Hz). The results of such studies have implications for the further development of sensory substitution devices and of specific rehabilitation programs for the hearing-impaired, who either use or do not use hearing aids or cochlear implants.

At this time, the nature of the final stage of STC hearing, i.e., the exact mechanism whereby the vibrations of the soft tissues which reach the ears and excite the inner ear hair cells is not clear. The inner ear is completely embedded in the densest bone of the body, the petrous bone. Therefore, it is not readily apparent how the soft tissue vibrations can “penetrate” into, and excite, the cochlea. However, while the 20-week-old fetus in utero is able to hear maternal sounds [17], it is completely surrounded by amniotic fluid, which also fills its middle ear cavity. Therefore, the fetus likely does not hear by an AC mechanism, since the amniotic fluid in the middle ear cavity would reduce the vibration of the middle ear ossicles. In addition, BC is also unlikely since the individual skull bones which comprise the skull are not yet fused, and membranous connective tissue sutures are situated between them [18] so that the skull bone cannot yet conduct vibrations, as would be required in order to elicit hearing by a BC mechanism. The mechanism which would explain the hearing of the fetus may also provide the basis for understanding the final mechanism of STC hearing in the adult. Clear assessment of the final stage of STC hearing may also contribute to better differentiation between STC and BC hearing, which is still considered a bit controversial [19,20]. In one of these previous studies [19], DPOAE was recorded in response to bone vibrator stimulation delivered directly on the dura mater in the course of craniotomy surgery, providing evidence that the STC pathway is direct and does not involve BC. Furthermore, given the two complementary attenuating factors (decrement in the magnitude of the soft tissue vibrations with distance as they are conducted toward the ear, coupled with the attenuation of the vibrations at the soft tissue–bone interface as a result of the higher acoustic impedance [21] of bone compared to that of the soft tissues), the threshold intensity for STC stimulation likely would not be able to induce vibrations of the skull bone at supra-threshold intensities. A similar STC study conducted in selected hearing-impaired patients may also contribute to a better understanding of the mechanisms involved and enhance the clinical relevance of STC. Vibration-based conduction pathways activated by a cartilage conduction hearing aid have also been recently investigated [22], and that study highlights the possible mechanisms, contributions and applications of the cartilage conduction pathway in the clinic.

The same research team that reported on “distantly-presented bone conduction perception” [3] was also able to use what they refer to as ultrasound stimulation (at 30k Hz) delivered to body sites on the neck, the clavicle and the upper limbs [23,24,25,26] in order to transmit speech information by using amplitude modulation. A preliminary report of the study [3] comparing audio-frequency to ultrasound stimulation was also presented [27]. Such stimulation was somehow able to elicit hearing thresholds, though the mechanism of such ultrasound hearing using amplitude modulation is unclear and requires further assessment. Also, use of the term “ultrasound” with respect to 30k Hz may be misleading. Instead, the phrase “beyond the frequency of the upper limit of human hearing” would be more appropriate. A future study should also assess the mechanism of what the authors refer to as the ultrasound hearing which they report in their published articles, [23,24,25,26], and how it can be applied to the transmission of speech information.

Study limitations: In the present study, STC sites on the cheek, neck and shoulder were stimulated, while in a previous study [2], sites more distant from the ear (down to thoracic vertebra 12-T_12_) were activated with 2k Hz stimulation, which is not within the frequency range of the cutaneous tactile mechanoreceptors, so that they were likely not involved. Furthermore, in the additional studies involving the delivery of vibratory stimulation to superior semi-circular canal dehiscence patients and normal control subjects [8,9], sites even as far from the ear as on the leg (ankle) were stimulated, though perhaps without adequate control for the possibility that their subjects may have been responding to AC sounds produced by the mini-shaker stimulator. In the future, it would be desirable to assess stimulation sites that are more dispersed and more distant on the body from the ear. To achieve this, it may be helpful to “shield” the bone vibrator in order to try to reduce the AC sounds which are also produced by the vibrator, which may reach the ear, and, in addition, by using ear muffs (protectors) over the ears, in addition to ear plugs in the external ear canal. In this way, it may be possible to assess STC stimulation sites that are more distant from the ear, thereby overcoming the “trade-off” between the distance of the stimulation site from the ear and the intensity of the stimulus required to excite the ear at a greater distance.

## Data Availability

The data that support the findings of this study are available on request from the corresponding author MG-D.
